# Cranial ultrasound by neonatologists

**DOI:** 10.1038/s41390-020-0779-8

**Published:** 2020-03-26

**Authors:** Paul Govaert, Charles C. Roehr, Pierre Gressens

**Affiliations:** 1 ZNA Middelheim, Antwerp, Belgium; 2 Newborn Services, John Radcliffe Hospital, Oxford University Hospitals NHS Foundation Trust, Oxford, UK; 3 Medical Sciences Division, Nuffield Department of Population Health, National Perinatal Epidemiology Unit, University of Oxford, Oxford, UK; 4 Université de Paris, NeuroDiderot, Inserm, Paris, France

This seminar is written by a group of neonatologists with a passion for cranial ultrasound (CUS) in the newborn. In many European neonatal intensive care units, CUS has been in the hands of the neonatologist for a few decades now. Although this gradually produced clinical knowledge and scientific production, apart from lecturing at symposia there is no formal transnational education with quality control in our group. This seminar, together with other efforts to transfer knowledge by bedside teaching (eurUS.brain), is part of a strive for formal guidelines and intercollegial assessment. It is greatly appreciated that this journal, together with the European Society of Pediatric Research, endorses support for education and science in the field of neonatology.

The brain of the newborn is not an organ where treatment options are actually abundant. Cooling has opened the box of possibilities, and several neuroprotective strategies are under investigation: the following decades will introduce strategies aiming at prevention and treatment of acquired brain lesions. To change management means we have to follow strict diagnostic rules both for inclusion in clinical trials and for individual care. That is one goal of this seminar. The second is to offer suggestions for research by challenging some outdated views on CUS. Throughout the work we carefully point to concepts that need validation. Cutting-edge technical opportunities are not included, because the aim was to summarize state-of-the-art CUS for common types of perinatal brain injury. This does not mean we do not hunger for attention by ultrasound vendors for the brain of the newborn. When appropriate, statements on CUS are compared with postmortem and MR findings of the disease at hand. These papers are produced in the regular fashion with a first author and co-authors, but members of eurUS.brain have contributed to all papers by final reading and correcting.

CUS is relatively complex because several windows to the brain can be used and different probes with variable settings are in vogue. The technical aspects of scanning, the natural part of training of radiologists, need to be brought under the attention of clinicians and this is done in the paper by Dudink et al.^1^. A complete careful scan in a fragile preterm infant is a piece of medical art, the action itself takes time and the report must be carefully written. Clinicians tend to conclude what diagnosis is most likely and what further diagnostic actions are potentially useful. Redirection of care is in their hands, often only upon careful evaluation of the extent of damage. A witticism of use here is that any CUS image or video framed should be of such quality to be usable in publications. Standard planes are indicated, avoiding to curtail the sonographer to so-called standard imaging, but encouraging exploration of additional views of any lesion or structure of interest. Routine scanning of a few sectional planes, as performed by lay people because it is part of the standard scheme, should become obsolete.

A systematic review, “Diagnostic and predictive value of Doppler ultrasound for evaluation of the brain circulation in preterm infants”,^2^ deals with the value of estimating arterial flow indices in preterm infants. The, not unexpected, salient finding is that this is an area where research did not offer strong evidence of anything. On the other hand, it is a paper that should encourage the reader to reconsider the future of CUS in studying brain perfusion, both at the macro- (arteries and veins) and microvascular level. The prognostic value of perfusion indices is a completely open field.

“Preterm germinal matrix hemorrhage, sequelae and outcome” was one of the next unavoidable topics. This is one of the injuries that remains prevalent. The paper by Parodi et al.^3^ discusses grading of the extent of this lesion paradigm. We should strive to completely prevent GMH after birth and to find ways of limiting evolving venous infarction near affected matrix areas. Given that the subventricular protomap of neuronal and glial progenitors is present in viable preterm infants, one can predict that the location and extent of matrix hemorrhage will correlate with specific dysfunctions. If we can predict such specificity, we will also find ways of mitigating dysfunction in survivors. MRI and CUS will be complementary in this topic.

Since we realized—by scanning via het mastoid fontanelle—that extremely low birthweight infants are at risk of cerebellar hemorrhage, “Ultrasound of acquired posterior fossa abnormalities in the newborn”^4^ became very actual. High-frequency linear probes provide access to many relevant lesions in the cerebellar hemispheres, but also to understanding transverse sinus thrombosis, the thrombotic heel of Achilles in preterms. Measurement of cerebellar size will be an important item in the prospective analysis of imperfect postnatal brain growth with CUS; this may define a subset of preterm infants’ candidate for targeted neuroprotection in a distant future. Not surprisingly, several other posterior fossa findings have caught our attention and are compared with MR findings.

Next to injury of germinal matrix (by germinolysis or hemorrhage) a prevalent type of injury is to white matter, both in preterm and term infants. “Preterm white matter injury: ultrasound diagnosis and classification”^5^ tackles the grading of white matter injury by CUS. As a group we felt this was perhaps the biggest challenge, because for years the acclaim has been that MRI was in fact the only reliable tool to study preterm white matter injury. In this chapter we demonstrate how specific injury types can be ascertained with CUS, and when combined with measurement of brain growth, how CUS studies can be planned that will offer prognostic insight, even in the absence of MR correlation. The periventricular white matter is also, because it is near the anterior fontanel, the area where we may expect improved diagnostic accuracy by technical advances of CUS, especially by the study of microvascular behavior. This is where vendors should embrace clinical research and offer bedside tools for objective measurement of tissue alteration with CUS. White matter perfusion monitoring with CUS is a direct research goal.

The original paper, “The development and validation of a cranial ultrasound scoring system for infants with hypoxic-ischemic encephalopathy”,^6^ concludes this seminar. It reviews the limited yet essential role of CUS in the diagnosis of perinatal asphyxia and propagates a scoring system that could be useful in situations where easy access to (repeated) MR scanning is not available.

As a network of neonatal CUS specialists, we realize that we have only just started. We are open to constructive suggestions from the pediatric as well as radiological community.

## Appendix: Members of eurUS.brain

Thais Agut, https://orcid.org/0000-0002-5673-0016

Ana Alarcon, https://orcid.org/0000-0001-7223-2028

Roberta Arena, https://orcid.org/0000-0002-8603-4206

Marco Bartocci, https://orcid.org/0000-0002-2454-6847

Mayka Bravo, https://orcid.org/0000-0002-2917-4288

Fernando Cabañas, https://orcid.org/0000-0002-1578-0703

Nuria Carreras, https://orcid.org/0000-0002-8770-2755

Olivier Claris

Jeroen Dudink, https://orcid.org/0000-0003-0446-3646

Monica Fumagalli, https://orcid.org/0000-0002-0186-0710

Paul Govaert, https://orcid.org/0000-0002-2011-4529

Sandra Horsch, https://orcid.org/0000-0002-2412-7319

Alessandro Parodi, https://orcid.org/0000-0002-3463-3004

Adelina Pellicer; https://orcid.org/0000-0001-8109-4958

Luca Ramenghi

Charles Roehr, https://orcid.org/0000-0001-7965-4637

Sylke Steggerda, https://orcid.org/0000-0002-5603-5697

Eva Valverde, https://orcid.org/0000-0002-2598-9172
